# Biodegradable Cell Microcarriers Based on Chitosan/Polyester Graft-Copolymers

**DOI:** 10.3390/molecules25081949

**Published:** 2020-04-22

**Authors:** Tatiana S. Demina, Maria G. Drozdova, Chantal Sevrin, Philippe Compère, Tatiana A. Akopova, Elena Markvicheva, Christian Grandfils

**Affiliations:** 1 Enikolopov Institute of Synthetic Polymeric Materials of Russian Academy of Sciences (ISPM RAS), 70 Profsoyuznaya str., 117393 Moscow, Russia; akopova@ispm.ru; 2 Institute for Regenerative Medicine, Sechenov First Moscow State Medical University (Sechenov University), 8–2 Trubetskaya str., 119991 Moscow, Russia; 3 Shemyakin & Ovchinnikov Institute of Bioorganic Chemistry, Russian Academy of Sciences, 16/10 Miklukho-Maklaya str., 117997 Moscow, Russia; drozdovamg@gmail.com (M.G.D.); lemark@ibch.ru (E.M.); 4 Interfaculty Research Centre on Biomaterials (CEIB), University of Liège, Chemistry Institute, B6C, 11 Allée du 6 août, B-4000 Liege (Sart-Tilman), Belgium; csevrin@ulg.ac.be (C.S.); pcompere@uliege.be (P.C.); c.grandfils@uliege.be (C.G.); 5 Centre for Applied Research and Education in Microscopy (CAREM), University of Liege, Chemistry Institute, B6C, 11 Allée du 6 août, B-4000 Liege (Sart-Tilman), Belgium

**Keywords:** microcarriers, graft-copolymers, oil/water emulsion, tissue engineering, fibroblasts, polylactide, chitosan

## Abstract

Self-stabilizing biodegradable microcarriers were produced via an oil/water solvent evaporation technique using amphiphilic chitosan-g-polyester copolymers as a core material in oil phase without the addition of any emulsifier in aqueous phase. The total yield of the copolymer-based microparticles reached up to 79 wt. %, which is comparable to a yield achievable using traditional emulsifiers. The kinetics of microparticle self-stabilization, monitored during their process, were correlated to the migration of hydrophilic copolymer’s moieties to the oil/water interface. With a favorable surface/volume ratio and the presence of bioadhesive natural fragments anchored to their surface, the performance of these novel microcarriers has been highlighted by evaluating cell morphology and proliferation within a week of cell cultivation in vitro.

## 1. Introduction

Cell microcarriers were first introduced in a mid-1960s for a large scale culture of anchorage-dependent cells in vitro for the production of several biopharmaceutical drugs [1,2]. For these applications, the microparticles have to fulfill a well-defined list of specifications, such as size, size distribution, morphology, density, and surface properties [3,4,5]. A shift to biodegradable cell carriers should allow their use as injectable scaffolds for regenerative medicine. It also opened a door to the concept of multifunctional cell carriers, whereby these particles could be loaded with bioactive components to enhance tissue reconstruction [6]. The specifications of these microcarriers should of course significantly differ compared to those established originally for in vitro biopharmaceutical drug production. Particularly, their surface morphology, biocompatibility, and biodegradability have to satisfy specific criteria, which are mostly dictated by their final clinical applications [7,8,9,10]. A wide variety of inorganic and organic materials have been already adopted as raw materials to prepare these microcarriers [7]. Amongst them, synthetic biodegradable aliphatic polyesters, such as polylactide, polyglycolide, poly(lactide-co-glycolide) (PLGA), etc. have received more attention [5,11,12,13]. They are indeed approved by U.S. Food and Drug Administration, are easy to process, and disclose good mechanical properties. However, these synthetic polymers do not provide any attractive binding sites to enhance animal cell adhesion. Complementarily, natural polysaccharides and proteins are well known to better promote animal cell adhesion through non-specific interactions (ionic force) or specific recognitions (adhesive peptide sequences specifically recognized by adhesion proteins expressed by animal cells) [14,15,16,17]. To enhance cell adhesion and growth onto polyester-based microspheres, surface post-modification is widely used [13,18]. This approach requires additional operational steps, such as preliminary surface activation/functionalization, bioactive component immobilization/deposition, washing out, etc., and the application of components, which could be potentially hazardous in terms of further application in biomedicine. Accordingly, the fabrication of microparticles made of a physical mixture of synthetic and natural polymers was described by Jiang et al. in [19], and by Yang et al. in [20] adopting a cellulose-graft-poly(l-lactide) copolymer. Recently, chitosan-g-oligo/polylactide copolymers were also adopted to prepare self-stabilized microspheres via the oil/water solvent evaporation technique. As a function of graft chain polymerization degree, the nature of polyester fragments, and chitosan characteristics, a set of microparticles with complex morphology was developed [21,22]. This former study evidenced that, above critical molecular weights of the hydrophilic chitosan backbone or of the hydrophobic polylactide moieties, the migration and self-assembly of these amphiphilic macromolecules at the oil/water interface was thwarted, resulting in a poor microparticle production yield. On the other hand, copolymers with short hydrophobic fragments led to the formation of brittle particles.

This study aimed to evaluate the fabrication’s features of self-stabilized microparticles via the oil/water technique using novel amphiphilic chitosan-g-polyester copolymers, and to assess them in vitro controlling animal cell adhesion and growth on their surface. To provide, along with better mechanical properties, a required flexibility to the macromolecules for their successful migration to the oil/water interface, amorphous poly(lactide-co-glycolide) moieties were grafted onto chitosan to produce an amphiphilic chitosan-g-PLGA copolymer (marked as CPLG). As a second option, an effect of the additional presence of hydrolyzed protein, i.e., gelatin, within the chitosan-g-poly(L-lactide) copolymer (the final terpolymer was marked as CPG) was studied in terms of the self-stabilization ability of formed microparticles and the further effect on their biocompatibility.

## 2. Results and Discussion

### 2.1. Microsphere Formation, Size Distribution and Recovery Yield

**Figure 1:**
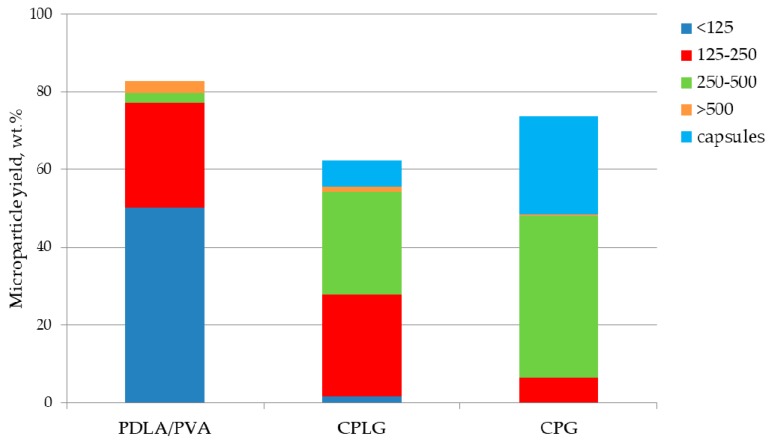
Effect of 30 wt. % replacement of PDLA in oil phase by CPG or CPLG copolymers on the processing yield and size distribution of the microspheres and capsule formation.

Amphiphilic chitosan-g-polyester copolymers, i.e., CPLG copolymer consisting of a chitosan backbone and grafted amorphous poly(lactide-co-glycolide) (PLGA) chains, and CPG copolymer comprising a chitosan backbone with grafted semi-crystalline poly(L-lactide) fragments and gelatin, were used as a core material for producing self-stabilizing biodegradable cell microcarriers. The detailed compositions and conditions of the synthesis of these copolymers are given in Section 3.1. For the sake of comparison with our former publication, we have assessed the copolymer’s interfacial properties to stabilize the oil/water emulsion and their miscibility with poly(D,L-lactide) (PDLA). This study was carried out adopting an oil phase made from a mixture of our graft-copolymer and PDLA (30/70 *w*/*w*) dissolved in methylene chloride. This screening study has highlighted that the two amphiphilic chitosan-g-polyester copolymers efficiently stabilize the oil/water interface in the absence of any additional emulsifier in the water phase. As disclosed in Figure 1 the microparticle recovery yield reached 62 and 74 wt. % for CPLG and CPG samples, respectively. Although slightly lower than observed for control poly(D,L-lactide) microparticles stabilized with polyvinyl alcohol (PVA), this material recovery is significantly higher than one observed for chitosan-g-poly(L,L-lactide) copolymers, i.e., 10 wt. % [22]. The majority of the copolymer microparticles have a size bigger than 200 μm, while the mean diameter of control microparticles is lower than 125 μm. The copolymer-based microspheres also have a larger particle size distribution than control microparticles.

Although this microsphere processing is well-known to be complex with the different physico-chemical events associated during the emulsification, evaporation, and hardening phases, the recovery yield and size distribution of microparticles are mostly governed by the stability and hardening kinetics of the oil/water interface. The kinetics of microsphere formation using traditional surfactants in aqueous phase is well-known to be controlled by several formulation parameters, such as processing temperature, agitation speed, nature of the oil and aqueous phases, and the macromolecular features of polymers used to generate the microparticles [23]. Adopting aliphatic polyesters and using PVA as an emulsifier, the phase separation leading to solid microparticles proceeds very quickly. Typically, the morphology and size of the microparticles are fixed within a time scale of some minutes after the onset of the oil/water dispersion [23].

**Figure 2:**
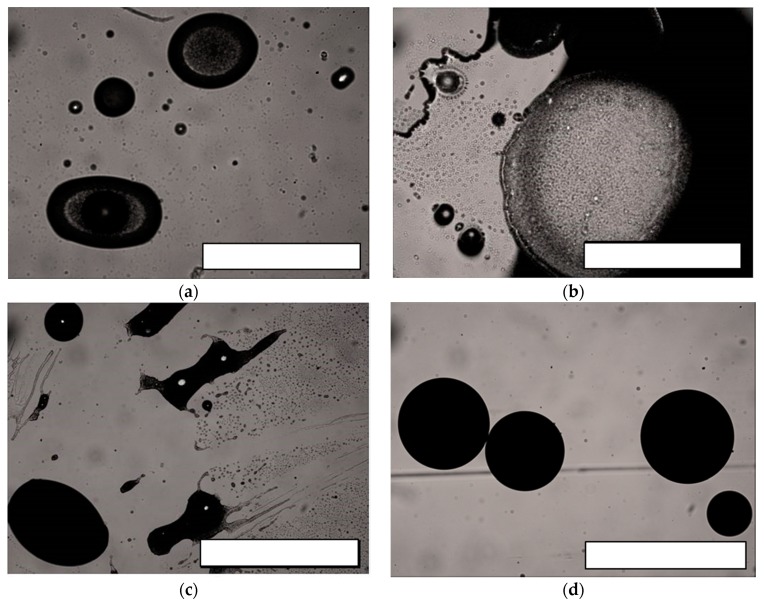
Micrographies acquired under optical transmission microscopes of aliquots of the oil/water emulsion containing CPG (10 wt. %)/PDLA in the oil phase and withdrawn at (**a**) 5 min, (**b**) 15 min, (**c**) 25 min and (**d**) 35 min after the dispersion onset. Scale bar is 1000 μm.

A kinetic study of microparticle interface stabilization was carried out by monitoring the evolution of the emulsions under optical transmission microscopy every 5 min. Interestingly enough, in the presence of a limited amount of the chitosan copolymers, i.e., 10 wt. % to PDLA dissolved in oil phase, the kinetics of microparticle formation were significantly slowed down. As highlighted from micrographies given in Figure 2, without PVA in the water phase, microparticle formation proceeded according to an unexpected and very slow evolution. A possible explanation could come from a combination of two events: a longer duration needed by our copolymers to migrate or/and change in conformation at the interface and their ability to limit the rate of solvent extraction to the continuous aqueous phase of the emulsion.

Indeed, up to 15 min, very few microparticles were detected. Instead, heterogeneous emulsion droplets with irregular shape were mostly visible under transmission microscopy. Further, after 25 min of emulsification, more distinct solid microparticles were identified, as disclosed on Figure 2c. It is only close to the end of the evaporation procedure, i.e., after 1 h, that solid spherical microparticles of a size range between 100 μm to 400 μm were observed. This extended duration needed to harden the polymer and to produce solid microparticles could be also explained by the limited solubility of our copolymers in the oil phase. As a consequence, we could anticipate that the stabilization of the oil/water emulsion and of the solid/liquid suspension after hardening could be mainly under the control of the slow kinetics of dissolution, migration, and rearrangement of our copolymers to these interfaces.

With an extended duration needed to stabilize and solidify the oil droplets, other physico-chemical events could occur, such as the coalescence or aggregation of oil droplets, but also foam production. Accordingly, these other interfaces could attract in a competitive way our copolymers. From the histogram given on Figure 1, a significant proportion of low density microparticles (capsules) have been recorded in the presence of the copolymers dissolved in the oil phase. These microparticles are characterized by a high floating ability due to the presence of significant amount of air bubbles entrapped inside the polymeric body. The formation of these bigger particles, with capsule inner structures with size up to 1.5 mm, has been already reported by Helou et al. [24]. Interestingly enough, these authors have noticed their formation with the adoption of amphiphilic polylactide-poly(ethylene oxide) block copolymers dissolved in oil phase.

**Figure 3:**
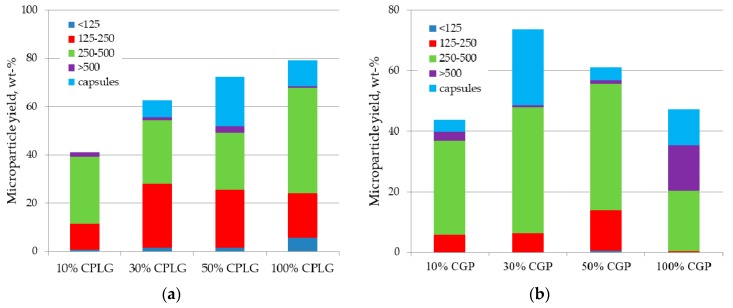
Effect of (**a**) CPLG or (**b**) CPG copolymers content in oil phase on the processing yield and size distribution of the microspheres.

The content of the copolymer in the oil phase, expressed in terms of wt. % to the total amount of polymers dissolved in the oil phase of the emulsion, has been also investigated to test their amphiphilic effectiveness and to verify the possibility to produce microparticles made only of these copolymers, thus free of any homopolyesters. Accordingly, the influence of the copolymer content, 10, 30, 50, and 100 wt. %, has been assessed for both copolymers measuring the total yield and size distribution of the resulting microspheres. As highlighted in Figure 3a, the increase in the wt proportion of chitosan-g-PLGA copolymer to PDLA dissolved in the oil phase improved the microsphere recovery yield. From a value of 41% noticed with a copolymer content of 10 wt. %, this recovery is progressively increased up to 80% when the oil phase is composed only of the graft-copolymer. As could be noticed from Figure 3, this increase in microparticle recovery is correlated with a progressive decrease in the mean size of the microparticles, an observation which supports the amphiphilic efficiency of the copolymer to stabilize the oil/water interface during processing.

This correlation between microparticle size and recovery yield is however more ambiguous with the terpolymer CPG, which highlighted a more complex behavior in terms of recovery yield and size distribution. Indeed, the mean microsphere size became lower up to 50% replacement of PDLA by CPG, while when microparticles were made from 100% CPG the proportion of particles above 500 µm is close to 50% (see Figure 3b).

**Figure 4:**
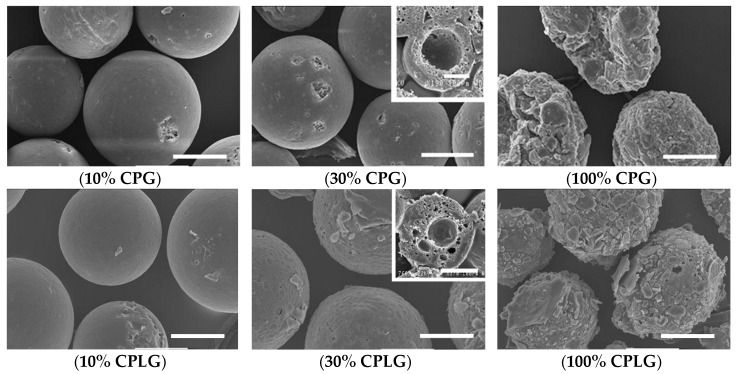
SEM micrographies of microparticles obtained by modifying the composition of the copolymer and its relative content in the oil phase of the o/w dispersion. The wt. % refers to the copolymer content to the total material in oil phase. Scale bars: 100 μm.

Expecting an influence of the amphiphilic copolymers on the surface of the microspheres, their morphology has been visualized under SEM microscopy. Compare to PDLA microspheres stabilized with PVA, which disclosed a smooth and regular surface, the copolymer microsphere roughness was gradually increased when increasing the copolymer content in the oil phase (Figure 4).

When made only of the copolymer, the surface roughness is not only affected, but the overall shape also became quite heterogeneous. According to these morphology observations, a 30 wt. % copolymer content could be considered as an “optimal” loading in order to achieve a maximum recovery yield without significantly affecting the surface roughness and cohesiveness of the microparticles. SEM observation showed a significant difference in morphology between the core and the surface of the particles (Figure 4 insertions). This difference could be linked to the amphiphilic nature of the copolymers and their tendency to generate other types of interfaces, such as an air/liquid one, leading to the formation of low-density particles including capsules. In contrast, the microparticle surface remains relatively regular, smooth, and non-porous. With a large roughness, this surface morphology could enhance cell adhesion [25,26]. A macroporous internal structure of the microspheres could be more favorable to facilitate microcarrier degradation and replacement with cells/tissue, due to a gradual increase of specific surface area when the microparticle shell is resorbed and the macroporous inner structure is available for cell adhesion.

**Figure 5:**
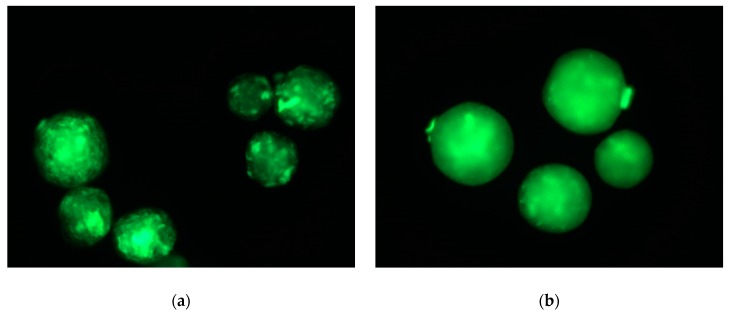
Micrographies of FITC-stained microparticles made from 30 wt. % of CPLG (**a**) and CPG (**b**) observed under fluorescence microscopy (Ex/Em 495/519 nm, respectively).

The migration of the graft-copolymer to the microsphere surface and the accessibility of the amino groups of chitosan or/and gelatin has been verified after fluorescein isothiocyanate (FITC) labelling and fluorescent microscopy. Although qualitative, this test should allow us to control the presence and accessibility of pending groups, well-known to affect adhesion of anchorage-dependent cells. As clearly highlighted in Figure 5, the FITC-staining of the microparticles evidenced a prominent fluorescent signal on the whole surface of the 30 wt. % of CPLG and CPG microparticles. These observations therefore prove the chemical accessibility and reactivity of amino groups of chitosan and gelatin after microparticle processing. The heterogeneity of the fluorescent signal could be related to a difference in inner porosity or to a local aggregation of chitosan moieties on the microparticle surface. A study of surface composition via the EDX analysis of copolymer-containing microparticles showed a considerable amount of nitrogen, even in the case of a 10 wt.% substitution of PDLA with CPG copolymer in oil phase. The N content was approx. 15-times higher than we could expect if the chitosan/gelatin moieties were randomly distributed over microparticle volume. Therefore, the EDX analysis confirmed that the natural copolymer moieties were migrated to the oil/water interface. These data are in a good agreement with the proposed mechanism of interface stabilization and the results of qualitative analysis of amino groups’ presence/accessibility via fluorescent labeling. Moreover, the amount of detected nitrogen was significantly varied (in a range of 5.45–6.74 at.%) as a function of spot on a copolymer-containing microparticle surface, suggesting a significant heterogeneity in surface composition. These data are well correlated with heterogeneity of the fluorescent signal (see Figure 5) and indicated a promising surface chemistry of the microparticles for their application as cell microcarriers.

The feasibility to produce microparticles in the absence of any common synthetic emulsifier in the aqueous phase make these amphiphilic copolymers particularly attractive for valorization. Indeed, the removal of stabilizer use allows to save several unit operations during the microparticle manufacturing, such as emulsifier dissolution, removal/washing out, and its regeneration/utilization. Moreover, in spite of the rather complex and slow kinetic of interface stabilization, these systems are very reproducible. The copolymers are especially promising to adopt them for the fabrication of microcarriers for tissue engineering. Indeed, in addition to their role in efficiently stabilizing the interfaces during processing, these biodegradable and natural made macromolecules provide binding moieties favorable to enhance cell adhesion.

### 2.2. In Vitro Analysis of the Biocompatibility of the Microparticles

**Figure 6:**
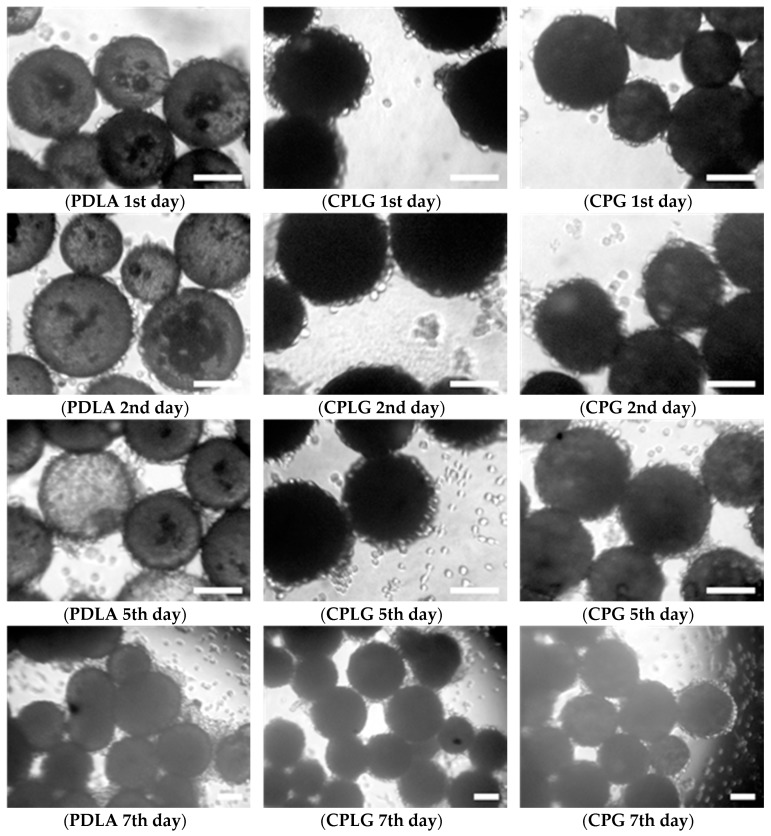
Microphotographies of L929 fibroblasts cultured on CPLG, CPG and PDLA microparticles. Scale bar is 100 μm.

The next step of the study was to evaluate animal cell interaction with these original microcarriers. In this study, L929 mouse fibroblasts were used as a model, taking into account that this cell line represents the reference for the evaluation of the cytotoxicity of medical devices according to ISO10093. This in vitro assay has been performed comparing PDLA-microparticles stabilized with PVA as a reference with the microparticles functionalized with our graft-copolymers. In order to assess the possible impact of the microparticles topography and chemistry on cell behavior, L929 fibroblasts were seeded and cultivated on the microbeads (Figure 6).

L929 fibroblasts were seeded on the microbeads and cultivated for seven days. In order to monitor cell behavior during in vitro cultivation, cell growth was observed under light microscopy. As noticed in Figure 6, the microcarriers efficiently supported cell adhesion and proliferation. After one day of incubation, several cells were already visible on the surface of the three types of microparticles. At day 2, most of the microparticle surface was covered with L929 fibroblasts, although they were mostly still well individualized with a relatively round sphere shape. From day 5 to day 7, a significant increase in cell density was observed with the formation of multilayers of cells covering the microcarriers.

Already at day 5, but especially on day 7, cross-linking of microcarriers occurred as a result of the multiplication of cells to the surface of two neighboring microcarriers, giving rise potentially to macroscopic cell/microcarrier aggregates. In all cases, thus independently of the microcarrier type or the duration of cell cultivation, free fibroblasts or fibroblasts adhering to polystyrene wells were noticed during our microscopic observations. This observation is not surprising taking into account the spontaneous sedimentation of these cells in the culture medium and the difficulty to maintain their homogeneous dispersion in the wells of this culture device. Although not quantitative, these observations do not highlight any significant difference in cell behavior, whatever the batch of microcarriers and the period of cell culture.

**Figure 7:**
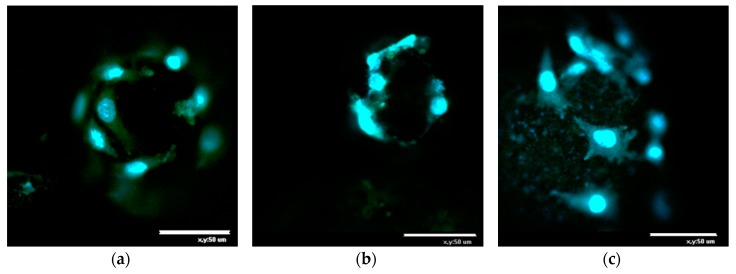
Confocal microphotographies of L929 fibroblasts after 3 days of culture on (**a**) control PDLA, 30 wt. % (**b**) CPLG- and (**c**) CPG-contained microparticles. Scale bar is 50 μm. Living cells were stained with vital fluorescent dye Calcein AM and cell nuclei were stained with DAPI.

In order to assess the viability of L929 fibroblasts cultivated on the microparticles, cells were stained with vital fluorescent dye Calcein AM and DAPI after three days of cultivation. The fluorescence micrographies reported in Figure 7 indicate that the three types of microcarriers promote cell adhesion and spreading on their surface. Moreover, taking into account that Calcein AM is converted to green fluorescent dye Calcein in living cells only, these latter observations demonstrate that those labelled fibroblasts were viable three days after adhesion and proliferation on these microbeads.

## 3. Materials and Methods

### 3.1. Copolymer’s Synthesis and Characterization

Amphiphilic chitosan-g-polyester copolymers were synthesized at ISPM RAS via the solid-state reactive co-extrusion of chitosan/poly(lactide-co-glycolide) blend (60/40 *w*/*w*) marked as CPLG and chitosan/poly-L-lactide/gelatin blend (35/52/13 *w*/*w*) (marked as CPG). Details of all copolymer synthesis batches and relative sample codes are given in Table 1. Low temperatures (above Tg, but below the melting points of the semi-crystalline polymers) are maintained throughout the process, minimizing the mechanical and oxidative degradation of the polymers. The obtained materials were characterized using fractionation, DLS, DSC, FTIR, and 1H NMR spectroscopy as previously published [27,28]. Commercially available poly(L-lactide) (Sigma) with an average Mw of 160 kDa and poly(D,L-lactide-co-glycolide) (Resomer, type 50:50, Boehringer Ingelheim) with Mw of 52 kDa were used throughout this study. Chitosan (Mw of 60 kDa; acetylation degree of 0.10) was prepared by the solid-state synthesis at ISPM RAS as reported earlier [29]. The grafting of polyester moieties onto chitosan chains was found to occur under the employed synthetic path, avoiding any catalysts, initiators, as well as organic solvents during processing, which allows to improve purity of the final materials. Taking into account an inevitable polydispersity of all initial polymers taken for the copolymer synthesis and a wide range of possible reactions (aminolysis or alcoholysis of any ester bonds by any chitosan functional groups), the copolymers consist of a wide set of macromolecular structures, differing in number and the length of grafted moieties. It was found that the employed technique leads to the substantial modification of the physico-chemical properties of the polysaccharide, in particular its propensity to disperse in organic solvents. According to DLS data, the materials produced at chosen conditions of blending form colloidal systems in chloroform with a mean size of the dispersed phase of 200–400 nm. This propensity of the obtained materials allows the manufacturing of microbeads with chitosan content up to 60 wt. % for the design of polyester-based biomaterials with improved bioactivity. Poly(D,L-lactide) (PDLA) (MW 76 kDa) was synthesized at CEIB and used as a part of the core polymer for microsphere preparation. Polyvinyl alcohol (PVA: 3-83, MW 18 kDa) used for fabrication of control PDLA microparticles was purchased from Hoechst (Germany). Fluorescein isothiocyanate (FITC) was purchased from Sigma-Aldrich. All other chemicals and solvents were of analytical grade and were used without additional purification.

### 3.2. Preparation and Characterization of the Microspheres

Microspheres were prepared by oil/water emulsion solvent evaporation technique as described previously [22]. The oil phase—8 wt. % solution of a copolymer or its mixture with PDLA in the mix solvent CH_2_Cl_2_/acetone (9/1 *v*/*v*)—was rapidly injected within an aqueous phase preliminary thermostatized at 15 °C under stirring using a 4-bladed propeller stirrer. The preparation of microspheres containing the copolymers in oil phase was carried out in mQ water, thus without any emulsifiers in the aqueous phase of this o/w emulsion. Control microspheres were made from PDLA as core polymer and a PVA solution (2.5% *w*/*w*) as aqueous phase to stabilize the oil/water emulsion. After the evaporation of organic solvents from the oil phase, the solid microspheres were carefully washed with mQ water, sieved with apertures ranging from 125 to 500 μm, and freeze-dried. The total yield of the microparticle production was calculated as wt. % of recovered microparticles to the total amount of polymer dissolved in oil phase. The microsphere size distribution was determined by sieving each microparticle batch using sieves of meshes ranging from 500 μm down to 125 μm in a wet mode. After fraction collection and drying, the weight of each particle fraction was determined by gravimetry. The mean yield of each microsphere batch was calculated as a weight percentage of recovered microspheres with given size to the total amount of polymer dissolved in oil phase. The data on total and fraction yields are given as a mean value calculated based on at least three separate processing runs; standard deviation ranged ± 3%.

The kinetics of microsphere formation was monitored under optical microscopic observation starting from 5 min after emulsification sampling the dispersions every 10 min. The microparticles were observed with an Olympus PROVIS AX70 in transmission mode equipped with a VisiCam 5.0 camera (VWR).

For classical scanning electron microscope (SEM) visualization of the microsphere surface and internal structure, microspheres were embedded within a concentrated PVA solution, dried, and cross-sectioned with a surgical blade. Both entire and sectioned microspheres were glued on a glass slide with double-sided carbon tape before being Pt-coated in a Balzers SCD 030 sputtering unit. Observations were carried out in a JEOL-840A (Japan) scanning electron microscope working at 20 kV accelerating voltage.

For environmental scanning electron microscopy (ESEM) and elemental X-ray microanalysis (EDX), the freeze-dried microspheres (without any coating) were viewed in ESEM-FEG XL-30 (FEI, Holland) with the gaseous secondary electron detector (GSE) in low vacuum condition (0.4 Torr) at 15 kV accelerating voltage. Images and position beam X-ray spectra were acquired with a silicon drift detector (Bruker SDD 10mm, 129eV) for light elements and analyzed through the Quantax Esprit 2.1. Semi-quantitative data in weight and atomic % were extracted from the spectra by use of the standard-less ZAF correction matrix after automatic back-ground subtraction.

The presence and accessibility of amino groups onto microparticle surface has been also verified using staining with FITC reagent as reported earlier using a Leica DFC-450 C (Leica Microsystems, Germany) fluorescent microscope [22].

### 3.3. Cell Cultivation on the Microparticles

The mouse fibroblasts (L929) were cultivated in DMEM medium supplemented with 10% FBS in flasks (25 cm^2^) in a CO_2_ incubator in 5% CO_2_ humidified atmosphere at 37 °C. The cells were re-seeded into a fresh medium every 2–4 days.

For the cell cultivation, dry microparticles were weighted, transferred into wells of 96-well plate, and sterilized with UV-irradiation (distance 5 cm) without cover of the plate for 2 h under agitation (speed 200 rpm). Microparticles after sterilization were washed three times with PBS and once with DMEM supplemented with 10% FBS. Cell cultivation with microcarriers was performed in non-adherent cell 96-well plates (Sarstedt, Germany). Each well contained 100 μL of cell suspension in culture medium (105 cells/mL) and 20 mg of microparticles.

Cell growth on the microcarriers was monitored using an inverted light microscope (Reichert Microstar 1820E, Germany) on days 1, 2, 5, and 7 of cultivation.

Cell morphology was evaluated on day 3 of cultivation using a confocal laser scanning microscope (CLSM) Nikon TE-2000 inverted microscope equipped with an EZ-C1 confocal laser (Nikon, Japan). After 3 days of cell cultivation, the cells were stained with Calcein AM and DNA fluorescent dyes (DAPI). For this purpose, the mixture of Calcein AM and DAPI in the medium (5 µg/mL of each dye) was added to the microcarrier samples, and the samples were incubated at 37 °C for 30 min. Then, the supernatant was replaced with fresh culture medium, and the samples were observed using a confocal laser microscope (Nikon TE-2000, Japan).

## 4. Conclusions

Amphiphilic chitosan-g-poly(lactide-co-glycolide) and chitosan-g-polylactide-g-gelatin copolymers were successfully used as core polymers for the fabrication of biodegradable cell microcarriers. The ability of these copolymers to stabilize the oil/water interface was highlighted and monitored during the microparticle formation via the oil/water solvent evaporation technique in the absence of any emulsifier in the aqueous phase. A complex copolymers’ chemical structure and kinetics of interface stabilization led to the formation of self-stabilized microspheres with a bigger mean size and a wider size distribution than ones produced with the aim of classical emulsifier (polyvinyl alcohol) in aqueous phase, while allowing to eliminate several manufacturing steps, such as emulsifier preparation, elimination, and regeneration/utilization. The microcarrier total yield, size distribution, and other characteristics, such as the porosity and roughness of volume/surface, have been shown to be easily modified by the modification of oil phase composition. Although no significant difference in fibroblast behavior was noticed between microcarrier batches and their surface properties, all of them were demonstrated to support efficient animal cell adhesion, spreading, and proliferation over a seven-day period. Moreover, the viability of these cells was demonstrated using green fluorescent dye Calcein staining.

## Figures and Tables

**Table 1:** Copolymer sample codes and synthesis details.

Sample Code	Component Content, wt.%				Temperature, °C
	chitosan	poly(L-lactide)	PLGA	gelatin	
CPLG	60	−	40	−	60
CPG	35	52	−	13	100
